# Dressing Head Wounds

**Published:** 1879-10-20

**Authors:** A. G. Smyth

**Affiliations:** Miss.


### *DRESSING HEAD WOUNDS.*

BY A. G. SMYTH, M.D., OF MISS.

In the August number of the Record is a short article on the treatment of wounds, "especially of the head." The writer has had what might be termed an extensive observation in the dressing of wounds of the head, such as are made by the kick of a horse, mule, or a blow with a stick. In cases where the wound is not lacerated, or with ragged or uneven edges, first, remove every trace of hair, blood or anything in the way. Stanch the bleeding, if possible; draw the wound together with isinglass plaster so as to close it completely. If thought to be necessary apply another covering of plaster. No bandage or other covering necessary, nor carbolic acid spray. If there is no secondary hemorrhage or deep suppuration, they almost always heal by first intention, leaving but little scar. No tearing out of sutures, no trouble with bacteria or atmospheric influence, so-called.

The same procedure is applicable to all wounds, no matter where situated, provided they can be kept dry, are not too extensive, or are liable from muscular motion to be torn asunder.

Your writer is of opinion that the whole subject is out of place and uncalled for; but as the subject of sutures and the question of carbolic spray are involved it may be necessary to show that the former can in many cases (they are always objectionable) be dispensed with. Also that the disagreeable accompaniments of the latter may be avoided without incurring the dangers from bacteria or atmospheric influence.

